# High expression of C-C chemokine receptor 2 associates with poor overall survival in gastric cancer patients after surgical resection

**DOI:** 10.18632/oncotarget.8069

**Published:** 2016-03-14

**Authors:** Ruochen Li, Heng Zhang, Hao Liu, Chao Lin, Yifan Cao, Weijuan Zhang, Zhenbin Shen, Jiejie Xu

**Affiliations:** ^1^ Department of Biochemistry and Molecular Biology, School of Basic Medical Sciences, Fudan University, Shanghai, China; ^2^ Department of General Surgery, Zhongshan Hospital, Fudan University, Shanghai, China; ^3^ Department of Immunology, School of Basic Medical Sciences, Fudan University, Shanghai, China

**Keywords:** gastric cancer, CCR2, overall survival, prognosis, biomarker

## Abstract

**Background:**

Being a critical chemokine receptor in chemoattracting myeloid cells into tumor tissues, C-C chemokine receptor 2 (CCR2) has been detected in many malignant tumors. This study aims to evaluate the prognostic value of CCR2 expression in patients with gastric cancer after surgery.

**Results:**

CCR2 expression was detected in the accessory cells around gastric cancer cells in a diffused manner. CCR2 high expression was correlated with tumor invasion depth (*P*=0.006 and *P*=0.004, respectively), lymph node metastasis (*P*=0.038 and *P*=0.011, respectively) and TNM stage (*P*=0.003 and *P*=0.001, respectively) in the two independent sets. Multivariate Cox regression analysis identifies CCR2 high expression was an independent poor prognostic factor for OS of patients with gastric cancer in the two sets (*P*=0.013 and *P*=0.006, respectively). Integration of CCR2 expression and TNM stage could provide additional prognostic value for OS than TNM stage alone in the two sets (*P*=0.038 and *P*=0.002, respectively).

**Methods:**

Two independent sets comprising a total of 474 patients who received standard gastrectomy were enrolled in the study. The expression level of CCR2 was detected by immunohistochemistry. The correlations between CCR2 expression and clinicopathological factors were explored, and the prognostic significance for overall survival (OS) was determined by Kaplan-Meier analysis.

**Conclusions:**

CCR2 high expression in the tumor microenvironment is a novel independent unfavorable prognostic factor for patients with gastric cancer. Combination of CCR2 expression and TNM stage could provide a better prognostic model for OS of gastric cancer patients.

## INTRODUCTION

Although the incidence and mortality of gastric cancer has decreased dramatically over the last 50 years in many regions [1], it still causes about 723,000 deaths per year around the world, making it the third leading cause of cancer related deaths after lung and liver cancer [2]. Owing to lack of specific symptoms at the early stage, a large number of gastric cancer patients were diagnosed at advanced stage and unsuitable for surgical resection, making it the main reason for poor prognosis [3]. Clinically used prognostic model for outcomes of gastric cancer patients mainly relies on tumor cell derived TNM stage [4]. However, the current prognostic model was unable to provide full prognostic information owing to not incorporating the information derived from the tumor microenvironment. Patients with the same TNM stage may have different outcomes owing to different tumor microenvironment [5]. Being the main cell type in constituting the tumor microenvironment, immune cells were important to determine a supportive or suppressive immune status for tumor cell growth and metastasis [6]. Thus, incorporating the tumor microenvironment derived prognostic information into TNM stage might provide better prognostic power.

Chemokine (C-C motif) receptor 2 (CCR2) is a 313 amino acid seven transmembrane G protein coupled receptor encoded on chromosome 3 and selectively binds the C-C chemokine CCL2 [7]. CCR2 was mainly expressed by the inflammatory cells in tissues including monocytes, dendritic cells, plasmacytoid dendritic cells and NK cells etc [8]. The activated CCR2 could mediate monocytes mobilization from the bone marrow and subsequent migration into target tissues [9], making it essential for effective control and clearance of infections, as well as pathogenesis of inflammatory, degenerative diseases and tumors [10].

Previous studies have demonstrated that CCR2 high expression in many malignant tumor tissues was positively correlated with disease progression and worse outcomes, including prostate cancer [11, 12], lung cancer [13], liver cancer [14], breast cancer [15], colorectal cancer [16] and pancreatic cancer [17]. However, contradictory results were obtained in ovarian cancer. *A. Sica et al* found CCR2 defection or relatively low expression was correlated with ovarian cancer progression, while others found CCR2 high expression promote tumor invasion [18–20]. Our previous study has identified that the main ligand for CCR2, CCL2, was highly expressed in gastric tumor tissues, and correlated with tumor progression [21]. But the expression profile of CCR2 in gastric cancer is largely unknown, and its relation with patient outcome remains obscure. Thus, detailed research on CCR2 expression in gastric cancer is urgently needed.

In this study, we aim to evaluate the expression profile of CCR2 in gastric cancer by immunohistochemistry (IHC), and investigate its correlation with clinicopathological factors and patient outcomes to refine the risk stratification system for predicting prognosis of patients with gastric cancer after surgical resection.

## RESULTS

### Expression of CCR2 shown by immunochemistry

**Figure 1:**
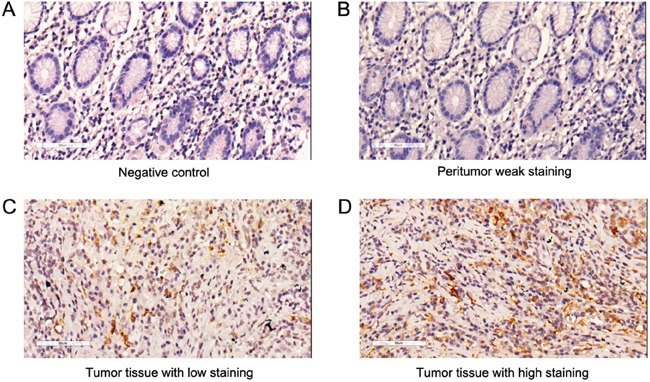
CCR2 expression in gastric cancer tissues and peritumoral tissues Representative photographs of CCR2 expression in gastric cancer tissue **A.** Negative control. **B.** Peritumoral weak staining. **C.** Tumor tissue with CCR2 low staining. **D.** Tumor tissue with CCR2 high staining. Original magnification: 200×.

In order to investigate CCR2 expression in gastric cancer and explore its potential clinical significance, we determined CCR2 expression levels by immunochemistry in a total of 474 gastric cancer patients with resectable tumor samples (96 in training set and 378 in validation set). The representative staining of CCR2 were shown in Figure 1. Tumor tissues showed more CCR2 staining compared to peritumoral normal tissues which was obtained from tumor resection margin. CCR2 expression was confined to the membrane of the accessory cells around gastric cancer cells in a diffused manner, while cancer cells showed negative staining. The numbers of positively stained cells within one view were used to signify the level of CCR2 expression and using the cut-off value determined by X-tile, 59.4% (57 of 96) and 48.9% (185of 378) were scored as low CCR2 expression in the training set and validation set, respectively.

### Relationship between CCR2 expression and clinicopathological parameters in gastric cancer patients

As shown in Table 1, CCR2 expression has positive correlation with tumor invasion depth (*P*=0.006 and *P*=0.004, respectively), lymph node metastasis (*P*=0.038 and *P*=0.011, respectively) and TNM stage (*P*=0.003 and *P*=0.001, respectively) in the two independent sets, while it has no significant correlation with gender, age, tumor differentiation and Lauren classification in the two sets. In addition, CCR2 expression was positively correlated with tumor size (*P*=0.042) and distant metastasis (*P*=0.028) in the validation set.

**Table 1:** Relationship between CCR2 expression and clinical characteristics in the training and validation sets of patients with gastric cancer

Factors	Training set					Validation set				
	Patients		CCR2 expression			Patients		CCR2 expression		
	*No.*	%	Low	High	*P*-value^a^	*No.*	%	Low	High	*P*-value
**All patients**	96	100	57	39		378	100	185	193	
**Age (years),**					0.599					0.052
Median (IQR)	60 (53-69)		60 (52-69)	60 (55-68)		60 (53-69)		61 (54-71)	59 (52-67)	
**Gender**					0.681					0.473
Female	37	38.5	21	16		114	30.2	59	55	
Male	59	61.5	36	23		264	69.8	126	138	
**Tumor size (cm),**					0.286					**0.042**
Median (IQR)	3.0 (2.0-4.8)		3.0 (2.0-4.0)	3.0 (2.0-5.0)		3.5 (2.0-5.0)		3.0 (2.0-5.0)	4.0 (2.5-5.0)	
**Differentiation**					0.346					0.085
Well	6	6.3	4	2		22	5.8	10	12	
Moderately	34	35.4	22	12		74	19.6	45	29	
Poorly	56	58.3	31	25		282	74.6	130	152	
**Lauren classification**					0.799					0.070
Intestinal type	71	74.0	43	28		230	63.0	125	105	
Diffuse type	25	26.0	14	11		148	37.0	60	88	
**T classification**					**0.006**					**0.004**
T1	29	30.2	22	7		66	17.5	39	27	
T2	10	10.4	9	1		55	14.5	34	21	
T3	4	4.2	1	3		66	17.5	31	35	
T4	53	55.2	25	28		191	50.5	81	110	
**N classification**					**0.038**					**0.011**
N0	41	42.7	30	11		144	38.1	80	64	
N1	18	18.8	8	10		39	10.4	21	18	
N2	14	14.6	8	6		77	20.4	37	40	
N3	23	24.0	11	12		118	31.1	47	71	
**Distant metastasis**					0.227					**0.028**
Absent	95	99.0	57	38		373	98.7	185	188	
Present	1	1.0	0	1		5	1.3	0	5	
**TNM stage**					**0.003**					**0.001**
I	33	34.4	26	7		91	24.1	55	36	
II	19	19.8	11	8		85	22.5	45	40	
III	43	44.8	20	23		197	52.1	85	112	
IV	1	1.0	0	1		5	1.3	0	5	

Abbreviations: TNM = tumor node metastasis; IQR = inter quartile range.

a *P*-value < 0.05 marked in bold font shows statistical significant.

### Prognostic value of CCR2 expression for overcall survival of gastric cancer patients

**Figure 2:**
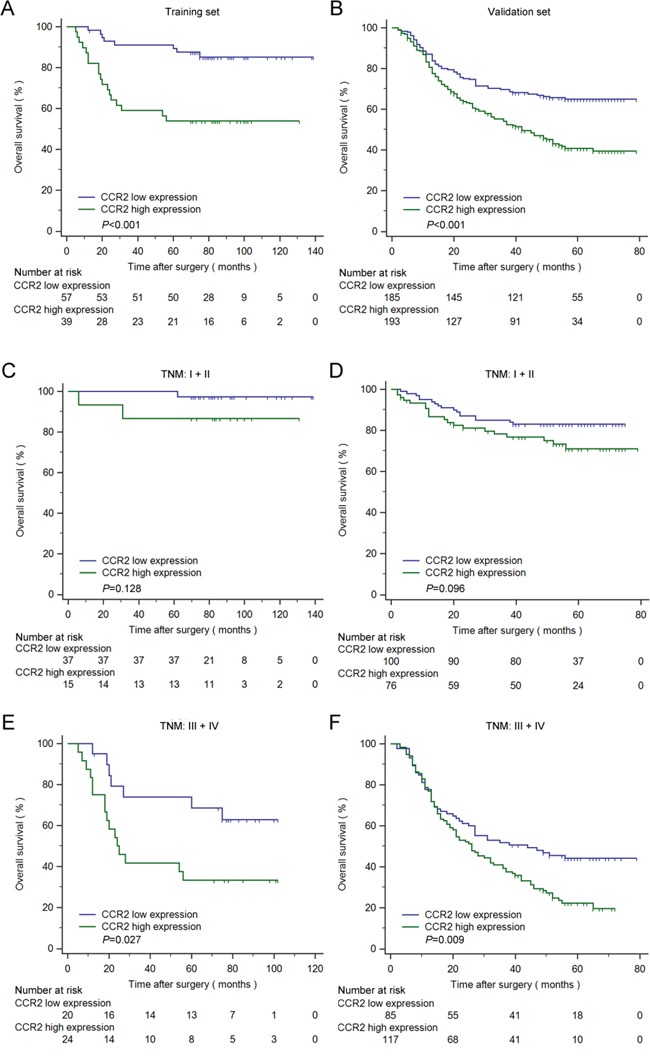
Kaplan–Meier analysis for OS of patients with gastric cancer according to CCR2 expression Kaplan–Meier analysis for OS of patients with gastric cancer according to CCR2 expression in all patients **A.** training set, n=96, *P*<0.001; **B.** validation set, n=378, *P*<0.001), TNM I+II **C.** training set, n=52, *P*=0.1276; **D.** validation set, n=176, *P*=0.0957) and TNM III+IV **E.** training set, n=44, *P*=0.027; **F.** validation set, n=202, *P*=0.009). *P*-value was calculated by log-rank test.

Kaplan–Meier survival analysis and log-rank test were applied to investigate the relationship between CCR2 expression and overall survival (OS) of patients with gastric cancer. Patients with CCR2 high expression shown shorter OS than those with CCR2 low expression in the training and validation set (Figure 2A and 2B, *P*<0.001 and *P*<0.001, respectively). To further determine whether CCR2 expression could stratify patients by TNM stage, we combined TNM I+II as early-stage disease while TNM III+IV as advanced-stage disease. Patients with CCR2 high expression in advanced-stage disease subgroups have shorter OS than those with CCR2 low expression in the training and validation set (Figure 2C and 2D, *P*=0.027 and *P*=0.009, respectively). However, in the early-stage disease, no statistically significant worse OS was found to be associated with high CCR2 expression, in spite of a decreasing tendency (Figure 2E and 2F, *P*=0.128 and 0.096, respectively).

**Figure 3:**
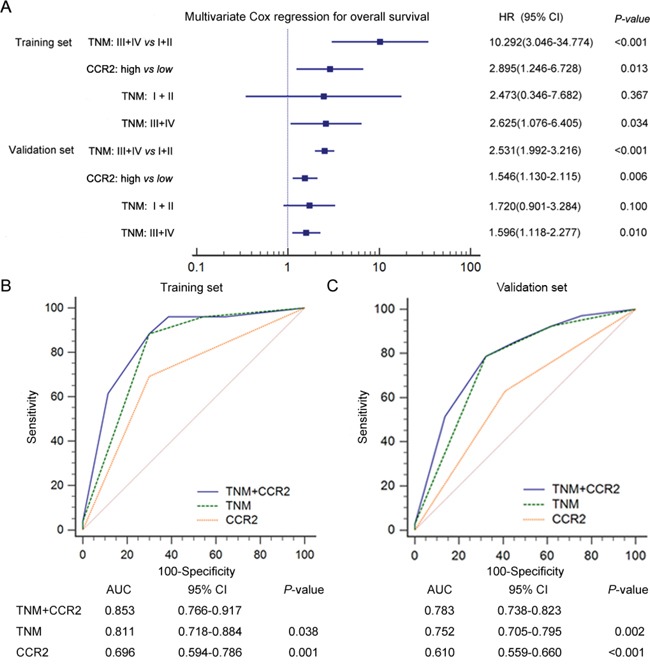
Multivariate Cox analysis and ROC analysis for prognostic accuracy of CCR2 expression in patients with gastric cancer **A.** Multivariate Cox analysis identified independent prognostic factors for the training and validation sets. ROC analysis of the sensitivity and specificity for the predictive value of TNM and CCR2 expression combined model, TNM model, CCR2 expression model in the training (B) and validation (C) sets. *P*-value <0.05 was considered statistically significant.

Univariate and multivariate analyses were performed to find prognostic factor for OS in patients with gastric cancer. As shown in Table 2, univariate analysis found that CCR2 expression (HR, 4.210; 95% CI, 1.828 to 9.700, *P*=0.001 and HR, 1.915; 95% CI, 1.405 to 2.609, *P*<0.001) along with T classification (HR, 13.652; 95% CI, 1.848 to 100.834, *P*=0.010 and HR, 4.819; 95% CI, 2.463 to 9.430, *P*<0.001), N classification (HR, 24.853; 95% CI, 3.364 to 183.625, *P*=0.002 and HR, 3.371; 95% CI, 2.295 to 4.593, *P*<0.001), distant metastasis (HR, 13.121; 95% CI, 1.614 to 106.645, *P*=0.016 and HR, 3.899; 95% CI, 1.591 to 9.552, *P*=0.003) and TNM stage (HR, 12.616; 95% CI, 3.776 to 42.148, *P*<0.001 and HR, 4.344; 95% CI, 3.016 to 6.258, *P*<0.001) were risk factors for OS in the training and validation sets respectively. To further estimate the independent prognostic value of CCR2 expression in gastric cancer patients, multivariate Cox regression analyses were performed by involving risk factors for OS derived from univariate analysis. TNM stage (HR, 10.292; 95% CI, 3.046-34.774, *P*<0.001 and HR, 2.531; 95% CI, 1.992-3.216, *P*<0.001) and CCR2 expression (HR, 2.895; 95% CI, 1.246-6.728, *P*=0.013 and HR, 1.546; 95% CI, 1.130-2.115, *P*=0.006) were identified as independent prognostic factors for OS in the training and validation sets, respectively (Figure 3A). In patients with advanced-stage disease, CCR2 high expression shown more risk for unfavorable OS (HR, 2.625; 95% CI, 1.076-6.405, *P*=0.034 and HR, 1.596; 95% CI, 1.118-2.227, *P*=0.010) while no such risk was found in patients with early-stage disease (HR, 2.473; 95% CI, 0.346-7.687, *P*=0.367 and HR, 1.720; 95% CI, 0.901-3.284, *P*=0.100) in the two independent sets, respectively. Taken together, these findings demonstrated that CCR2 high expression could be an independent poor prognosticator for patients with gastric cancer, especially for patients with advanced-stage disease.

**Table 2:** Univariate Cox regression analyses for overall survival in the training and validation sets of patients with gastric cancer

Factors	Overall Survival					
	Training set			Validation set		
	HR	95%CI	*P*-value a	HR	95%CI	*P*-value
**Age (years)**	1.022	0.985-1.060	0.257	1.017	1.002-1.029	**0.012**
**Differentiation:** poorly *vs* moderately + well	1.453	0.648-3.261	0.365	1.485	1.020-2.161	**0.039**
**Lauren classification:** diffuse *vs* intestinal	1.054	0.443-2.509	0.905	1.274	0.942-1.725	0.116
**Tumor size (cm)**	1.122	0.930-1.253	0.230	1.090	1.023-1.162	**0.008**
**T classification:** T2+T3+T4 *vs* T1	13.652	1.848-100.834	**0.010**	4.819	2.463-9.430	**<0.001**
**N classification:** N1+N2+N3 *vs* N0	24.853	3.364-183.625	**0.002**	3.371	2.295-4.953	**<0.001**
**Distant metastasis:** present *vs* absent	13.121	1.614-106.645	**0.016**	3.899	1.591-9.552	**0.003**
**TNM stage:** III+IV *vs* I+II	12.616	3.776-42.148	**<0.001**	4.344	3.016-6.258	**<0.001**
**CCR2 expression:** high *vs* low	4.210	1.828-9.700	**0.001**	1.915	1.405-2.609	**<0.001**

Abbreviation: 95% CI= 95% confidence interval; HR= hazard ratio; TNM=tumor node metastasis.

a *P*-value<0.05 marked in bold font shows statistically significant.

### Improvement of the TNM staging prognostic model with CCR2 expression

To improve the prognostic accuracy for OS of patients with gastric cancer, we combined CCR2 expression and TNM staging system to generate a predictive model. Receiver Operating Characteristic (ROC) analysis was applied to compare the prognostic accuracy between CCR2 expression + TNM staging system or TNM staging system alone. We found that the combination of CCR2 expression and TNM staging system showed significantly higher prognostic accuracy (AUC 0.853, 95% CI 0.766 to 0.917 and AUC 0.783, 95% CI 0.738 to 0.823) than CCR2 expression alone (AUC 0.696, 95% CI 0.594 to 0.786, *P*=0.0010 and AUC 0.610, 95% CI 0.559 to 0.660, *P*<0.0001) or TNM staging system alone (AUC 0.811, 95% CI 0.718 to 0.884, *P*=0.038 and AUC 0.752, 95% CI 0.705 to 0.795, *P*=0.002) in both training and validation sets, respectively (Figure 3B and 3C). All these results indicated that the combined CCR2 expression and TNM staging system model show more prognostic power for OS of patients with gastric cancer.

## DISCUSSION

Traditional TNM staging system for predicting outcomes of patients with gastric cancer mainly relies on information derived from tumor cell phenotype, such as tumor cell invasion depth, lymph node metastasis and distant metastasis [23]. However, current TNM prognostic system failed to incorporate the prognostic information derived from tumor microenvironment. More and more studies have found the accessory cells around tumor cells could constitute a tumor promotive or suppressive microenvironment to participate tumor biology. In our present study, we found that CCR2 was highly expressed on the accessory cells around gastric cancer cells and correlated with tumor stage. Furthermore, the expression level of CCR2 could be used to stratify patients with different outcomes, especially in patients with advanced III/IV disease. Multivariate Cox regression analysis identified CCR2 high expression in gastric cancer tissues is an independent poor prognostic factor for patients with gastric cancer. All these results need a larger, multicenter dataset to validate.

CCR2 has been widely studied since its discovery of role in mediating the recruitment and migration of monocytes into the target sites along with its major ligands gradient, which was secreted by both tumor and stromal cells [24]. Tumor associated macrophages (TAMs), the most abundant immune cells infiltrating into tumor tissues, have been found to be an important kind of cells in mediating host immune reaction to tumor cells with a polarized function role (M1, tumor suppressive phenotype; M2, tumor promotive phenotype) according to tumor microenvironment. TAMs can be attracted into tumor microenvironment by cytokines or chemokines released by tumor cells or other accessory cells. Previous studies have demonstrated that CCR2 could stimulate macrophage trafficking and induced a M2 phenotype skew. The M2 skewed TAMs could promote tumor progression by promoting angiogenesis and lymphangiogenesis and our previous study has identified the prognostic significance of polarized macrophages infiltration in patients with gastric cancer. Thus, we speculate that [25–34] the CCR2 high expression in tumor microenvironment was an indicator for high M2 infiltration and correlated with tumor progression.

Along with TAMs, Myeloid-derived suppressor cells (MDSCs), which comprised immature myeloid progenitors for neutrophils, monocytes and dendritic cells, were also a heterogenous population of myeloid lineage immune cells that enhance tumor progression [35]. MDSCs could promote tumor growth not only by modulating immune responses towards tumor tolerance, but also by supporting several processes necessary for the neoplastic progression such as tumor angiogenesis, cancer stemness, and metastasis dissemination [36, 37]. Notably, the migration to tumor of MDSCs depended mainly on CCL2/CCR2 pathway [38]. More recently study revealed that, mesenchymal stem-like cells(MSLCs), the progenitor cells of fibroblast and adipocyte, which could migrate to tumor sites and then promote tumor growth, were also recruited by CCL2/CCR2 pathway [39]. Another article clarified that CCR2 participates in the recruitment of bone marrow-derived fibroblasts into the kidney during the development of renal fibrosis, thus we speculated CCR2 may promote tumor development by recruiting some bone marrow-derived fibroblasts into the tumor sites and then become cancer associated fibroblasts(CAF) in the tumor microenvironment [40]. Therefore, the CCR2 positive cells in tumor tissues may TAMs, MDSCs, MSLCs or some bone morrow-derived fibroblasts. And all these evidences suggest that CCR2 may facilitate gastric cancer progression by recruiting immune suppressive cells into tumor microenvironment to create a tumor-promotive microenvironment.

Our present study has proved the prognostic significance of CCR2 expression in patients with gastric cancer, and refined the risk stratification system which based on TNM stage alone. In addition to being a potential prognostic factor for many malignancies, CCR2 might receive considerable attention as an effective marker for predicting therapeutic outcomes and is a potential target for anti-cancer therapy. Targeting CCR2 could decreased tumor-initiating cells, relieves immunosuppression and improves chemotherapeutic effect in mice pancreatic cancer [17, 41]. Besides, an antagonist of CCR2 could abolish alcohol-stimulated migration or growth in colorectal and breast cancer [42, 43]. All these studies have demonstrated the blockage of CCR2 may have therapeutic effect in malignant tumors. But the profound molecular roles of CCR2 and its antagonist in gastric cancer remains far from being fully elucidated and need further research.

Although we have proved the prognostic significance of CCR2 expression in patients with gastric cancer, especially in those with advanced-stage disease, there are some limitations in our study. We detected the expression of CCR2 by means of IHC, which was somewhat subjective. Recurrence free survival (RFS) was not analyzed owing to lack of RFS data. Moreover, the study was retrospectively designed in nature. A large, multi-center, prospective data is needed to validate the results.

In summary, our results suggested that CCR2 high expression independently predicts poor postoperative overall survival for patients with gastric cancer. Incorporation of CCR2 expression with TNM stage system might add some prognostic information for patients with gastric cancer. Furthermore, CCR2 might become a potential immunotherapeutic target for gastric cancer.

## MATERIALS AND METHODS

### Patients and follow-up

Two independent sets comprising a total of 474 patients who received standard gastrectomy with lymphadenectomy by the same surgical team in Zhongshan Hospital were enrolled in our study. 468 patients received primary tumor resection, while one patient in the training set and the five patients in the validation set with resectable liver metastasis were also received metastatic tumor resection. The training set comprising 96 consecutive patients was recruited between 2000 and 2005, while the validation set comprising 378 consecutive patients was recruited during 2008. The use of human specimens was approved by the Clinical Research Ethics Committee of Zhongshan Hospital, and written informed consent from each patient was achieved. Patients with large areas of necrotic and hemorrhagic samples or without detailed follow-up data were excluded. Clinicopathological information including age, gender, tumor location, tumor size, tumor differentiation, Lauren classification, and tumor stage was obtained for each patient from medical record. Overall survival was defined as the time from the date of surgery to the date of death or last visit. All the patients were followed up until April 2014 with the median follow-up time of 78 and 47 months in training and validation set, respectively.

### Tissue microarray construction and immunohistochemistry

Tissue microarray construction was performed as previous described [22]. Primary Anti-CCR2 antibody (diluted 1:500; Abcam, Cambridge, UK) was used for IHC staining. Computerized image system composed of an Olympus CCD camera connected to a Nikon eclipse Ti-s microscope was used to measure the density of positive staining. The IHC staining sections were scanned at high-power magnification (×200) and captured by NIS-Elements F3.2 software to identify the five random independent microscopic fields to ensure representativeness and homogeneity. Identical parameters were used for each photograph. The evaluation of immunostaining was performed by two independent gastroenterology pathologists who were blinded to the patient outcomes and clinicopathological characteristics. The numbers of positively stained cells within one view were used to signify the level of CCR2 expression, and nine was determined as the cut-off value on the basis of a minimum *P*-value approach calculated by X-tile software (Yale University, New Haven, CT).

### Statistical analysis

Analyses were performed with SPSS 21.0 (IBM, Armonk, NY), MedCalc Software version 12.7.0 (MedCalc, Mariakerke, Belgium) and Stata 12.0 (Stata CorpLP, College Station, TX). Pearson χ^2^ test or Fisher's exact test was used to compare categorical variables, and numerical variables were analyzed by the *t* test or Pearson's correlation test. Kaplan-Meier analysis was used to determine the survival. Log-rank test was used to compare patient survival between subgroups. The stepwise Cox proportional hazard regression model was used to perform univariate and multivariate analyses. Numbers at risk were calculated for the beginning of each time period. Receiver operating characteristic (ROC) analysis were used to compare the accuracy of the prediction of clinical outcome by the parameters. All *P*-values were two-sided, and differences were considered significant at values of *P*<0.05.
